# Development and Validation of Rapid Colorimetric Reverse Transcription Loop-Mediated Isothermal Amplification for Detection of Rift Valley Fever Virus

**DOI:** 10.1155/2023/1863980

**Published:** 2023-01-30

**Authors:** Francis Wekesa, Mark Wamalwa, Richard Oduor, Yatinder Binepal, Leonard Ateya, Noah Okumu, Angela M'kwenda, Christopher Masaba, Eugine Mukhaye

**Affiliations:** ^1^Department of Biochemistry, Microbiology and Biotechnology, Kenyatta University, P.O. Box 43844-00100, Nairobi, Kenya; ^2^Kenya Agricultural Livestock and Research Organization, Biotechnology Research Institute, P.O. Box 57811-00100, Nairobi, Kenya

## Abstract

Rift Valley fever virus (RVFV) is a high-priority zoonotic pathogen with the ability to cause massive loss during its outbreak within a very short period of time. Lack of a highly sensitive, instant reading diagnostic method for RVFV, which is more suitable for on-site testing, is a big gap that needs to be addressed. The aim of this study was to develop a novel one-step reverse transcription loop-mediated isothermal amplification (RT-LAMP) method for the rapid detection of RVFV. To achieve this, the selected RVFV *M* segment nucleotide sequences were aligned using Multiple Sequence Comparison by Log-Expectation (MUSCLE) software in MEGA11 version 11.0.11 program to identify conserved regions. A 211 pb sequence was identified and six different primers to amplify it were designed using NEB LAMP Primer design tool version 1.1.0. The specificity of the designed primers was tested using primer BLAST, and a primer set, specific to RVFV and able to form a loop, was selected. In this study, we developed a single-tube test based on calorimetric RT-LAMP that enabled the visual detection of RVFV within 30 minutes at 65°C. Diagnostic sensitivity and specificity of the newly developed kit were compared with RVFV qRT-PCR, using total RNA samples extracted from 118 blood samples. The colorimetric RT-LAMP assay had a sensitivity of 98.36% and a specificity of 96.49%. The developed RT-LAMP was found to be tenfold more sensitive compared to the RVFV qRT-PCR assay commonly used in the confirmatory diagnosis of RVFV.

## 1. Introduction

Rift Valley fever (RVF) is a transmissible zoonotic disease caused by the Rift Valley fever Virus (RVFV) [1]. During an outbreak, the disease spreads rapidly irrespective of the national border, causing serious socioeconomic and public health consequences [2, 3]. The infection is characterized by massive abortions in pregnant animals and death among the animal population with mortality rates as high as 90% in young animals and 30% in adults [4]. This leads to a drastic loss of herds and flocks, resulting in food insecurity and loss of revenue not only to the farmers but also to the traders, butchers, and the affected country [5]. The disease also causes death among the naive human population with ever-increasing severity with a mortality rate of up to 28% [6].

The RVFV belongs to the Phlebovirus genus in the family Phenuviridae [7]. It is an enveloped, tri-segmented, and negative-stranded RNA [8]. Its genome consists of three segments, large (*L*), medium (*M*), and small (*S*), based on their molecular size [9, 10]. The *L* segment encodes a viral RNA-dependent RNA polymerase (Large protein) [11]. The *M* segment encodes two glycoproteins, Gn and Gc that form the viral envelope, and two nonstructural proteins, NSm and Gn/NSm fusion proteins [8, 12]. Finally, the *S* segment encodes a nucleoprotein gene, NC, and a nonstructural protein gene, NSs, in sense and antisense orientations, respectively [13].

Laboratory diagnosis of RVFV currently relies on enzyme-linked immunosorbent assay (ELISA), viral neutralization tests (VNT), reverse transcriptase polymerase chain reaction (RT-PCR), and real-time RT-PCR (qRT-PCR) [14]. However, besides being cumbersome, the ELISA and VNT have shown cross-reactivity with antibodies of cocirculating members of other closely related phleboviruses, and they also require biocontainment or laboratory level 3 facilities and highly qualified personnel to carry out [15]. On the other hand, molecular assays, like RT-PCR and qRT-PCR, require sophisticated and well-equipped laboratories with well-trained personnel [16]. These restrict their application in terms of resources and capabilities in many developing countries, with Kenya being no exception.

This study exploited LAMP technology in the development and evaluation of the sensitivity and specificity of a rapid colorimetric RVFV RT-LAMP assay targeting the M RNA segment of RVFV. The LAMP technology has been successfully employed in the development of diagnostic kits against dengue virus, Indian citrus ringspot virus, fowl adenovirus, Japanese encephalitis virus, filarial parasites, *Talaromyces flavus* species among others [17–22]. This is because the RT-LAMP method offers an inexpensive and simple-to-use point-of-care simple diagnostic assay that is highly accurate, sensitive, flexible, and easy to scale up during an outbreak [23–25].

## 2. Materials and Methods

### 2.1. Study Area

This study was conducted at the KALRO Biotechnology Research Center, Kabete, Nairobi, Kenya.

### 2.2. Primer Design

**Figure 1:**
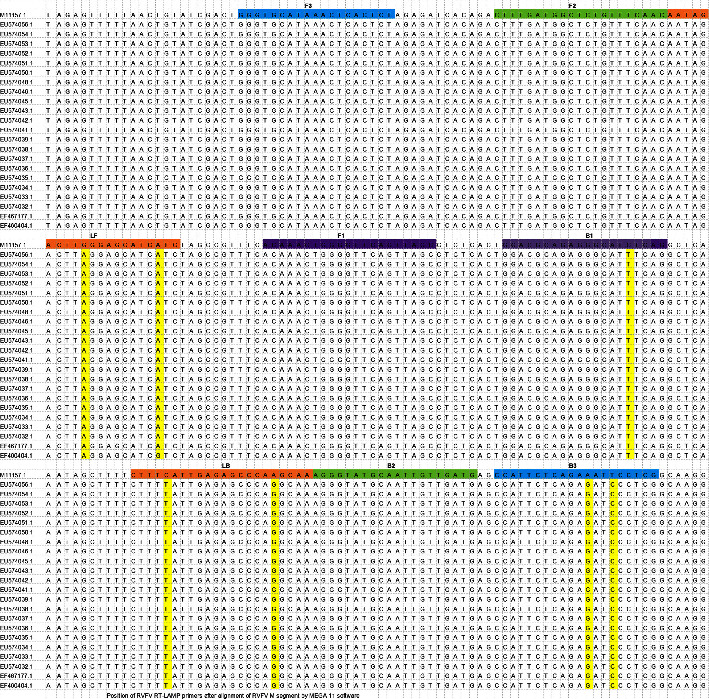
Position of RVFV RT-LAMP assay primers after alignment of RVFV *M* segment using MEGA 11 version 11.0.11 software.

Twenty-three complete sequences of the RVFV M segment from the NCBI database were aligned as previously described using Multiple Sequence Comparison by Log-Expectation (MUSCLE) software in MEGA11 version 11.0.11 program to identify conserved regions [26] (Tamura *et al.*, 2021). The potential target region of 211 bp corresponding to the RVFV M segment genome sequence position 2584–2794 of the RVFV prototype ZH-501 (accession number M11157.1) was used as the template against which the RT-LAMP primers were designed. A total of six primers were designed, two outer primers (F3 and B3), two inner primers (FIP and BIP), and two loop primers (LF and LB) (Figure 1). The primers targeted eight distinct positions of the template and were designed using NEB LAMP Primer design tool version 1.1.0 (New England Biolabs) under default settings. The specificity of the designed primers against RVFV was determined using the nucleotide-based Basic Local Alignment Search Tool (BLASTn). Only primer sets that were highly specific to the RVFV strain, able to form a loop, and with melting temperatures below 65°C were selected.

### 2.3. Rift Valley Fever Virus Strain (RVFV)

In this study, frozen RVFV seed stock strains RVF VIR 1983, RVF VIR G64, RVF VIR 66, RVF 5335, and RVF OVI vaccine maintained in Baby Hamster Kidney (BHK) cell lines and preserved by 90% glycerol were used. The strains were donated by Dr. Yatinder Singh Binepal, the head of Livestock Biotechnology at KALRO Biotechnology Center, Kabete, Nairobi, Kenya.

### 2.4. Cell Culture

The study employed the use of Vero cells isolated from kidney epithelial cells of African green monkeys passaged in Roswell Park Memorial Institute (RPMI) 1640 medium (ThermoFisher Scientific, Waltham, USA) supplemented with 10% of sterile fetal bovine serum (FBS) (ThermoFisher Scientific, Waltham, USA) as previously described by Ammerman et al. [27]. This was carried out by slight modification to include RPMI 1640 growth medium instead of DMEM medium. The cells were observed daily for confluence and contamination. On the fourth day, the cells formed over 90% monolayer confluence and were used for viral replication.

## 3. Infection of Vero Cells with RVFV

The frozen RVFV seed stocks labeled RVF VIR 1983, RVF VIR G64, RVF VIR 66, RVF 5335, and OVI vaccine were used to infect a confluent monolayer of Vero cells as previously described by Smith et al. [28]. The RPMI suspension medium was used instead of DMEM medium as previously described [28]. Two negative controls, media-only and Vero cell-only flasks, were included to check for contamination and comparison of cytopathic effect (CPE). The flasks were observed daily for four days, and the flasks showing more than 90% CPE characteristic of Rift Valley fever virus were selected for total RNA extraction.

### 3.1. Extraction and Purification of RNA from the RVFV Strain

Total RNA was extracted from the infected cell monolayer using the TRIzol Reagent method as described by the manufacturer (Cat. No. 15596026, Invitrogen Life Technologies, Waltham, USA). Briefly, the suspension medium was carefully decanted, and the Vero cell monolayers from both infected and uninfected flasks were used for total RNA extraction. The cells were lysed by adding 4 mL of TRIzol Reagent to each flask on ice. Lysis was achieved by pipetting up and down until the monolayer was fully broken down. 1 mL of the lysate was aseptically transferred into clearly labeled 1.5 mL Eppendorf tubes and incubated at room temperature for 5 min to allow complete dissociation of the nucleoprotein complex. Phase separation was realized by the addition of 0.2 ml chloroform to each tube containing the lysate and mixing vigorously for 15 seconds. This was followed by incubation at 25°C for 3 minutes and spinning at 12000 rpm for 15 min at +4°C to separate the mixture into three phases, the aqueous upper phase, the interphase, and the lower organic phase. The upper phase, carrying total RNA, was carefully transferred to a sterile 1.5 mL Eppendorf tube without disturbing the interphase. Thereafter, 0.5 mL of cold isopropyl alcohol was added and the tubes were incubated at −20°C overnight to allow precipitation of the total RNA. The tubes were then centrifuged at 12000 rpm for 10 min at +4°C, the supernatant discarded, and the pellet washed by the addition of 70% ethanol. Washing was achieved by a brief suspension of the pellet in wash buffer (70% ethanol) followed by spinning at 7500 rpm for 5 min. The supernatant was discarded, and the pellet was air-dried for 10 min. The pellet was finally eluted in 50 *µ*L of molecular-grade water. The purity of the extracted total RNA was determined by the ratio A260/A280, and the concentration was determined by measuring the OD at A260 using a Nanodrop 2000c spectrophotometer (Cat. No. ND-2000, ThermoFisher Scientific, Waltham, USA). The extracted RNA from both the RVFV-infected and -uninfected cells was stored at −40°C.

### 3.2. Reverse Transcriptase Polymerase Chain Reaction (RTPCR)

The RVFV total RNA extracts were subjected to reverse transcriptase polymerase chain reaction (RTPCR) using previously designed glycoprotein specific primers. The 5″ATAGAATTCAAGGAGATGCCACCTTGTCG3′ and 5′TATGGATCCTGTGGGCAGAGAGAGATCCA3′ were used as forward and reverse primers, respectively. The amplification of the RVFV glycoprotein gene was optimized and used a one-step RTPCR kit combining both the superscript III reverse transcriptase (RT) and high-fidelity platinum Taq polymerase according to the manufacturer's protocol (Cat. No. 2574026, Invitrogen, ThermoFisher Scientific, Waltham, USA). Briefly, a 50 *µ*L reaction volume was prepared in a sterile 0.2 mL sterile PCR tube on an ice block by mixing 25 *µ*L of 2X reaction buffer, 2 *µ*L of the total RNA template, 1 *µ*L of each primer (forward and reverse primers), 2 *µ*L of the enzyme mix (Superscript RT/Platinum Taq mix), and the volume adjusted to 50 *µ*L using molecular grade-distilled water. The contents were mixed by pipetting up and down, followed by a brief spin to collect them at the bottom of the PCR tubes. RT-PCR was carried out in a highly sensitive Veriti 96-well thermocycler (Cat. No. 4375305, Applied Biosystems, ThermoFisher Scientific, Waltham, USA) at 45°C for 30 min, 94°C for 4 min, followed by 35 cycles of 94°C for 30 seconds, 57°C for 30 seconds, and 72°C for 1 min. A final extension was done at 72°C for 10 min before holding at 4°C until agarose gel analysis. The confirmation of the presence of RVFV was done by running the RTPCR products on a 1% agarose gel electrophoresis with a standard 1 kb molecular weight ladder (Invitrogen). Clear and distinct bands with the correct sizes were an indication of the presence of the RNA from RVFV. Nontemplate control and RNA from noninfected Vero cells were used as negative controls.

### 3.3. Real-Time Reverse Transcriptase Polymerase Chain Reaction (qRT-PCR)

The total RNA extracted from RVFV strains RVF VIR 1983, RVF VIR G64, RVF VIR 66, RVF 5335, and OVI vaccine from infected Vero cells were used. The primers VFL-2912fwdGG(5′-TGAAAATTCCTGAGACACATGG-3′),RVFL-2981revAC(5′-ACTTCCTTGCATCATCTGATG-3′), and probe; RVFL-probe-2950 (5′-FAM-CAATGTAAGGGGCCTGTGTGGACTTGTG-BHQ1-3′) from CDC Atlanta targeting the RVFV *L* segment was used during the qRT–PCR as previously described by Bird et al. [29]. Briefly, 12.5 *µ*L of 2*x* universal master mix (Applied Biosystem, ThermoFisher Scientific, Waltham, USA), 0.5 *µ*L of each primer, 0.5 *µ*L of the probe, 5 *µ*L of total RNA, 1 *µ*L of enzyme Mix, and 5 *µ*L of nuclease-freemolecular-grade water were mixed in a 25 *µ*L reaction volume. The amplification and cycling parameters for one-stepqRT-PCR were 45°C for 10 min, 95°C for 15 min, 40 cycles of 95°C for 0.25 min, and 60°C for 1 min as cycling parameters in QuantStudio™ version 5 thermocycler (Cat. No. 437305, ThermoFisher Scientific, Waltham, USA). Nontemplate and noninfected Vero cell RNA tubes were used as negative controls.

### 3.4. Standard Curve for RVFV Using qRT-PCR

The efficiency of the qRT-PCR as a reference diagnostic test was established by determining the values of *R*^2^ and the slope of the RVFV standard curve as described in the QuantStudio™ version 5 user manual. Briefly, a positive sample with a known concentration of RVFV total RNA was diluted to five dilution points at a ratio of 1 : 10. The concentration of total RNA was expressed in pg/*µ*L starting from the highest to the lowest concentration. The master mix for qRT-PCR was prepared according to the protocol previously described by Bird et al. [29]. Briefly, master mixes for 15 standard samples and 3 negative control samples were prepared by mixing 237.5 *µ*L of 2X RTPCR buffer, 19 *µ*L of RVFVL F/R primers, 9.5 *µ*L of the RVFVL probe, 19 *µ*L of 25X RTPCR enzyme, and 171 *µ*L of molecular-gradenuclease-free water and mixed by pipetting up and down. The 24 *µ*L of the master mix was dispensed into each of the 0.2 mL sterile PCR tubes and 1 *µ*L of each sample was added accordingly. The samples were spun briefly to collect the content at the bottom of the PCR tubes. For accuracy and reproducibility, this test was carried out in triplicate. The standard curve protocol was set up as described by the QuantStudio™ version 5 thermocycler manufacturer (Cat. No. 437305, ThermoFisher Scientific, Waltham, USA) using default settings and the cycling conditions.

### 3.5. Optimization of the Reaction Conditions of the Colorimetric-RVFV-UDG-RT-LAMP Assay

This was done as previously described by Poole et al. [19] with a slight modification to include WarmStart® Multipurpose LAMP/RT-LAMP 2X Master Mix (with UDG) Protocol (Cat. No. M1708, Biolabs Inc, New England, UK). Briefly, a 25 *µ*L LAMP reaction volume was prepared by mixing 12.5 *µ*L of WarmStart® Multipurpose LAMP/RT-LAMP 2X Master Mix (with UDG), 2.5 *µ*L 10*X* primer mix, 0.1 *µ*L indicator dye, and 2 *µ*L of extracted RNA. The pH was adjusted to 8.2–8.6 using potassium hydroxide. The total volume was adjusted to 25 *µ*L using sterile molecular-grade water. A nontemplate control (NTC) tube was used as a negative control. The RVFV-UDG-RT-LAMP reaction was performed at different temperatures (50, 55, 60, and 65°C) at different periods (30, 45, and 60 min) in a Veriti 96-well thermocycler (Cat. No. 437305, ThermoFisher Scientific, Waltham, USA). The reaction was terminated by heating at 80°C for 5 min. Amplicons were evaluated visually by observing color changes and confirmed through gel electrophoresis on a 1% agarose gel stained with ethidium bromide dye. The impact of loop primers (LP) on the RT-LAMP was assessed at the optimized temperature by running two sets of experiments, one with LP and the other without LP at different periods (30, 45, and 60 min) at 65°C. For accuracy and repeatability, each experiment was done in triplicate and the results were analyzed by running 5 *µ*L of each product on 1% agarose gel electrophoresis stained with ethidium bromide and observed under ultraviolet light.

### 3.6. Monitoring of Colorimetric-RVFV-UDG-RT-LAMP Amplification

Successful colorimetric RVFV-UDG-RT-LAMP reactions were monitored through naked eye visualization and confirmed through agarose gel electrophoresis analysis. Naked-eye visualization was achieved in two ways, mainly through color change from pink to yellow by the inclusion of 0.1 *µ*L phenol red as a pH indicator to RT-LAMP reaction reagents or by observation of a white precipitate after a brief spin of the final product after RT-LAMP assay. As a confirmation, agarose gel electrophoresis analysis was carried out by running 5 *µ*L of the RVFV-UDGRT-LAMP products on 1% agarose gel stained with ethidium bromide in 1% Tris-borate buffer (TBE) and visualized UV in a gel Doc™ EZ imager version 5.1 (Cat. No. 735BR06006, Bio-Rad, California, USA).

### 3.7. Specificity of the Colorimetric-RVFV-UDG-RT-LAMP Assay

To test the specificity, total RNA extracted from Vero cells infected with different strains of RVFV (G66, 1983, 5335, and G64) as well as from two other major pathogens of small ruminants (Peste des petits ruminants (PPR) and Capripox viruses) were used. The RVFV-UDG-RT-LAMP reaction was performed using 1 ng of the total RNA extracted as the template under optimized conditions for both temperature and duration, as described earlier in this study. The results were visualized by observation of color changes and confirmed through agarose gel electrophoresis as previously described.

### 3.8. Evaluation of Analytical Sensitivity of the Colorimetric-RVFV-UDG-RT-LAMP Assay

The analytical sensitivity of the RT-LAMP assay was assessed by comparing the optimized RVFV-UDG-RT-LAMP assay with real-time RT-PCR by performing a tenfold serial dilution of the known concentration of RVFV total RNA with an initial concentration of 1.85 × 10^6^ pg/*µ*L as determined using a Nanodrop 2000c spectrophotometer ((Cat. No. ND-2000, ThermoFisher Scientific, Waltham, USA). Nontemplate control and total RNA from noninfected Vero cells were used as negative controls. The colorimetric-RVFV-UDG-RT-LAMP assay protocol was performed on all dilutions under optimized conditions and its outcomes were compared with those of qRT-PCR. Successful RT-LAMP assay amplification was analyzed visually based on color changes and/or by agarose gel electrophoresis, while the threshold cycle (Ct) value between 8 and 35 was regarded as positive by qRT-PCR. The sensitivity for the colorimetric RVFV-RT-LAMP assay and qRT-PCR was defined as the final dilution that yielded positive amplification.

### 3.9. Validation of the Colorimetric-RVFV-UDG-RT-LAMP Assay Using Clinical Samples

The newly developed colorimetric-RVFV-UDG-RT-LAMP assay was validated under optimized conditions (63°C for 30 min) using total RNA extracted from 118 reference field samples suspected to be infected with RVFV obtained with permission from the Directorate of Veterinary Services (DVS). The 118 field samples were purposefully selected after initial screening using IgM capture ELISA (61 IgM positive blood samples and 57 IgM negative blood samples) as previously described by Ellis et al. [30]. Total RNA was extracted from each sample using MagMAX™-96 Viral RNA Isolation Kit according to the manufacturer's protocol (Cat. No. AM1836, ThermoFisher Scientific, Waltham, USA). Briefly, 50 *µ*L of blood sample was dispensed into each well on the processing plate and clearly labeled. Then, 20 *µ*L of bead mix and 130 *µ*L Lysis/Binding Solution was added to each well-containing sample and mixed by shaking at maximum speed for 3 min to allow lysis of viral cells and binding of RNA to the binding beads. The binding beads carrying the total RNA were magnetically captured by allowing the 96-well processing plate to stand for 1 min on 96-WellMagnetic-Ring Stands. The supernatant was carefully discarded without disturbing the beads by aspiration. The processing plate was removed from the magnetic stand and washed twice using 150 *µ*L of wash solution 1. This was followed by washing with 150 *µ*L wash solution 2 twice. The RNA binding beads with RNA were then recaptured on a magnetic stand as in the previous step and the supernatant was aspirated. The total RNA was eluted by the addition of 50 *µ*L of elution buffer to each sample and shaking for 3 min at maximum speed. The RNA binding beads were captured as outlined in the previous steps and the supernatant containing the RNA was carefully transferred to a clearly labeled RNase-free Applied Biosystems microamp 96 PCR plate (Cat. No 10124183, ThermoFisher Scientific, Waltham, USA) and stored at −20°C. The extracted RNA was then subjected to RVFV qRT–PCR and colorimetric-RVFV-UDG-RT-LAMP assays as described previously, and the results were compared. The results were termed positive when the Ct value was between 8 and 35 for the qRT-PCR and the color change from pink to yellow for the colorimetric-RVFV-UDG-RT-LAMP assay.

## 4. Data Analysis

Mega 11 version 11.0.11 was used to align selected M segment sequences of RVFV strains to determine highly conserved regions. A one-way ANOVA was used to determine whether there was a statistically significant difference between the means of three independent groups of total RNA extracted using TRIzol Reagent using IBM SPSS statistics version 28.0.0.0 (IBM corporation). For statistically significant data, the Tukey post-hoc test was used to compare the mean between each pairwise combination of groups to determine which groups were different from each other. For statistically insignificant data, the group means and standard deviation (SD) were recorded.

## 5. Results

**Figure 2:**
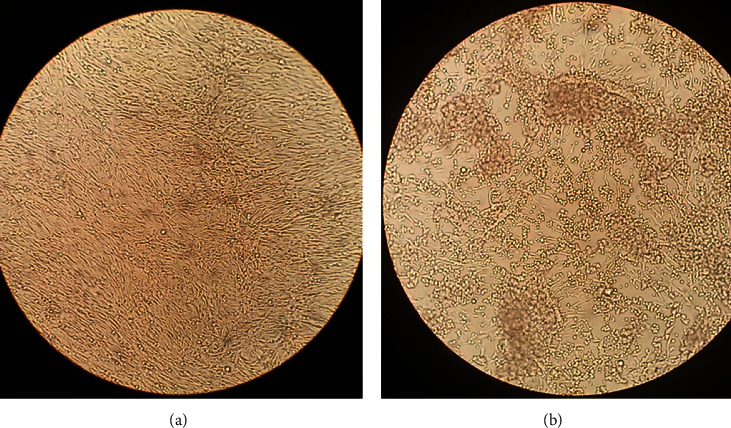
Vero cells as viewed under an inverted microscope (Leitz Labovert), (a) Vero cells without RVFV and (b) Vero cells infected with RVFV after four days.

**Figure 3:**
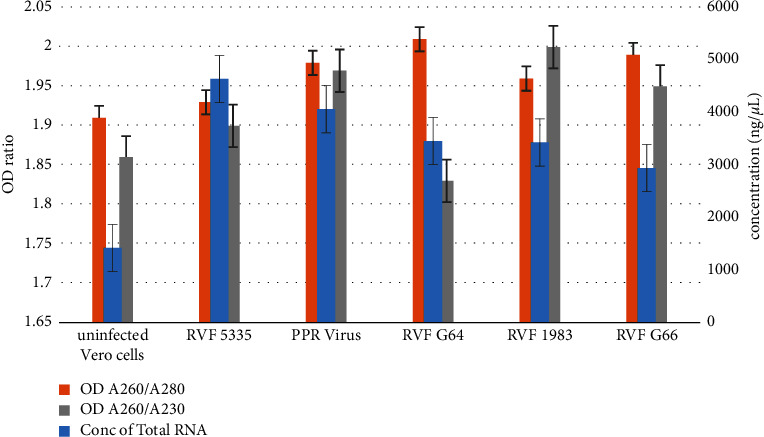
Concentration of total RNA (ng/*µ*L), OD ratios of 260/280, and 260/230 of extracted total RNA by Nanodrop 2000c spectrophotometry. The bars stand for the standard error (*n* = 3).

The RVF viral strains were successfully replicated in Vero cells. The rounding up of more than 90% Vero cells on the fourth day after RVFV infection compared with noninfected Vero cells (negative control) was a clear indication of RVF viral replication (Figure 2). Infection of Vero cells was performed in a replica of three and total RNA from both infected and noninfected Vero cells was extracted using TRIzol Reagent. There was no statistically significant difference in the concentration and purity of total RNA extracted from the three replica groups as determined using one-way ANOVA (*F* (2, 15) = 0.000187, *p*=0.999). The mean ± standard deviation of the concentration and purity of total RNA extracted from the RVFV-infected and noninfected Vero cells were recorded (Table 1 and Figure 3).

**Figure 4:**
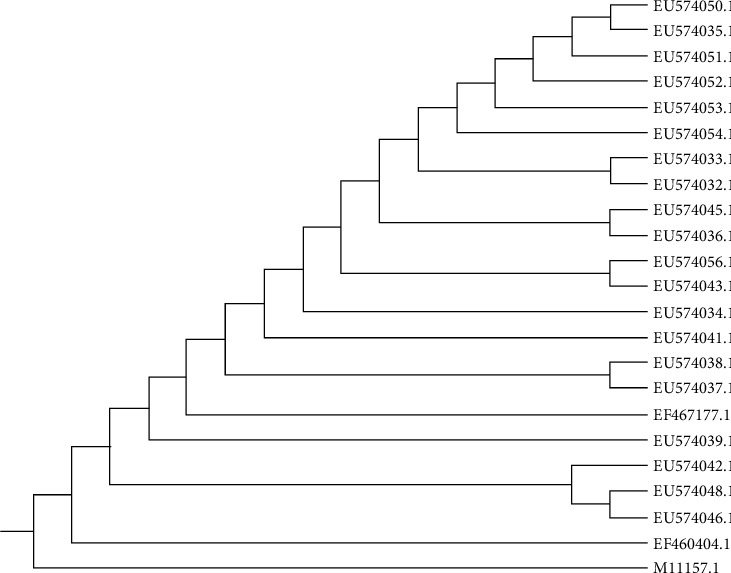
Genetic relationship of the RVFV strains used during the alignment of *M* segment sequences using the MEGA 11 version 11.0.11 tree explorer program.

**Figure 5:**
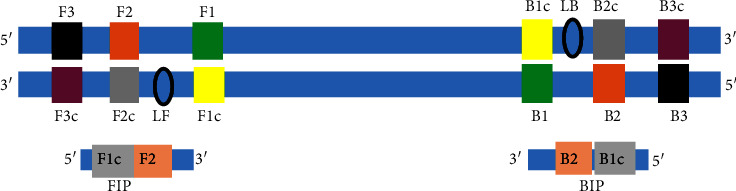
Schematic representation of the primers designed for the RT-LAMP assay. **F3** is the forward external primer, **B3** is the backward external primer, **FIP** is the forward inner primer, **BIP** is the backward inner primer, **LF** is the loop forward primer, LB is the loop backward primer, **B1c** is the complementary sequence of B1, and **F1c** is the complementary sequence of F1. The inner primers **FIP** and **BIP** with both sense and antisense sequences allow the formation of a loop.

The twenty-three RVFV *M* segment sequences whose genetic diversity is shown in Figure 4 were aligned and a highly conserved region of 211 bp was identified (Figure 1). Six sets of primers were designed and their locations on the template sequence are indicated in Figure 1 and Table 2. The primers were found to be specific to different strains of RVFV around the world. The relationship of the primers to one another is schematically shown in Figure 5.

**Figure 6:**
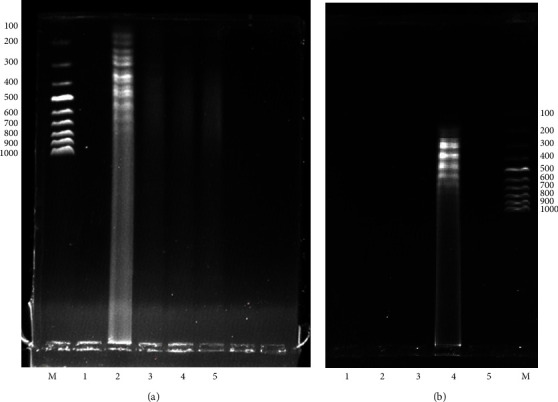
Optimization of optimum conditions for RT-LAMP. (a) Optimization of the amplification temperatures for the RT-LAMP assay M-100 bp molecular ladder, **1**-NTC, **2**-65°C, **3**-60°C, **4**-55°C, and **5**-50°C for a duration of one hour. (b) Optimization of the incubation duration for the RT-LAMP assay was **1**–**50** minutes, **2**–**55** minutes, **3**–**60** minutes, and **4**–**65** minutes, M-100 bp molecular ladder at 65°C.

**Figure 7:**
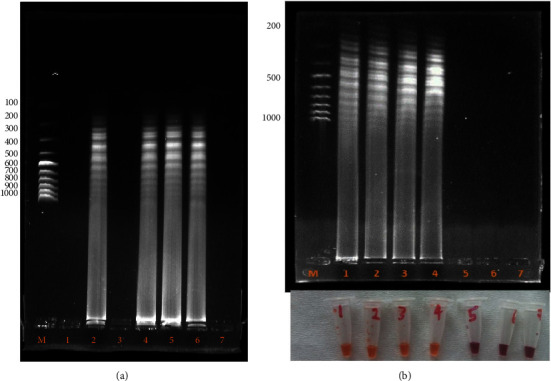
(a) Effect of loop primers (LP) on sensitivity and incubation duration. M-molecular ladder, **1‒30** minutes without LP; **2‒30** minutes with LP; **3‒45** minutes without LP; **4**–**45** minutes with LP; **5**–**60** minutes without LP; and **6**–**60** minutes with LP. (b) Specificity of the colorimetric-RVFV-UDG-RT-LAMP assay. M-100 bp molecular ladder, **1**–**4** RVFV strains G66, 1983, 5335, and G64, **5**-PPR, **6**-Capripox, **7**-NTC.

**Figure 8:**
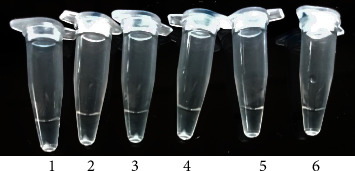
Determination of the final amplification products for the RVFV-UDG-RT-LAMP by observation of white precipitate. **1**-RVF 5335, **2-**RVF G64, **3**-RVF-1984, **4**-RVF-64, **5-**PPR, and **6**-NTC.

**Figure 9:**
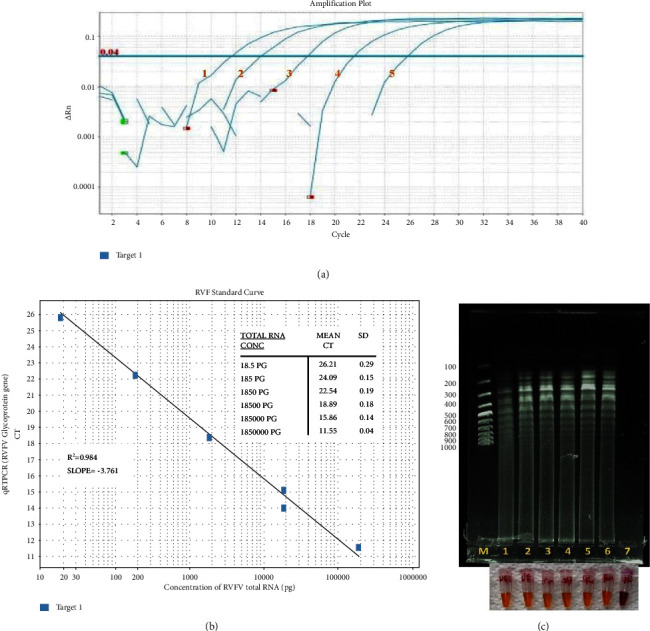
Detection limit of the new colorimetric-RVFV-UDG-RT-LAMP assay. (a) Analytical sensitivity of qRT-PCR of six replicates determined by tenfold dilution of known concentration of RVFV total RNA (18.5 pg–1850000 pg). (b) Standard curve generated by plotting the number of RVFV RNA copies (*x*-axis), and the mean of the corresponding qRT–PCR threshold cycle (Ct) of three independent experiments on the *y*-axis. (c) Limit of detection of the RVFV-UDG-RT-LAMP assay using a tenfold dilution of known concentration of RVFV total RNA as described under qRT-PCR as monitored through color change and resolved on 1% agarose gel electrophoresis. The M-100 bp molecular ladder (Cat SM0241, Thermo Fisher Scientific Inc., USA), **7-NTC**, **1**–**6** tenfold dilution from 18.5 pg to 1.85 × 10^6^, respectively.

**Figure 10:**
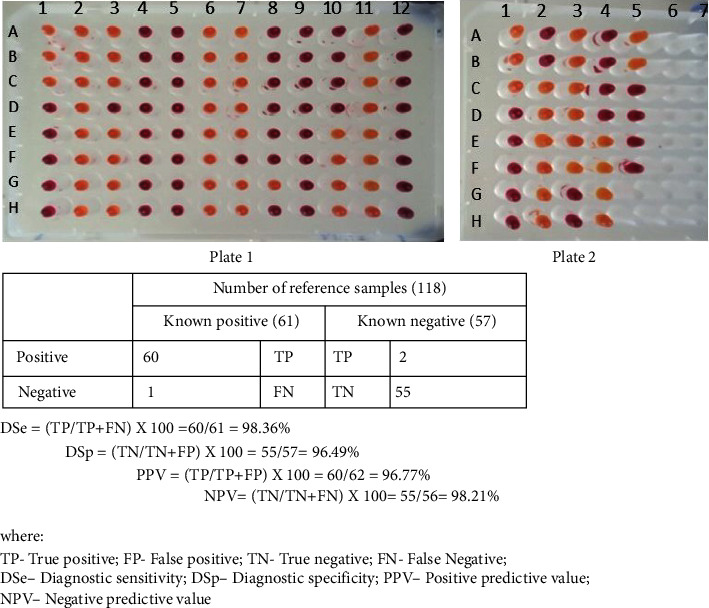
Determination of analytical sensitivity, specificity, positive predictive value, and negative predictive value for the colorimetric-RVFV-UDG-RT-lamp assay compared with qRT-PCR and IgM capture ELISA.

To optimize the amplification temperature for the RVFV RT-LAMP assay, the total RNA from RVFV-infected Vero cells was used as the template at different temperatures based on previous studies (50, 55, 60, and 65°C) for 60 min. The results revealed that the optimum amplification temperature was achieved at 65°C (Figure 6(a)). To determine the optimum amplification time, the RNA template was amplified for different durations (30, 45, and 60 minutes) at 65°C. The optimum amplification time was 60 minutes (Figure 6(b)). The determination of the final amplification products for the RVFV-UDG-RT-LAMP was carried out by visualization using the naked eye through the observation of a white precipitate, color change from pink to yellow, and ladder-like bands on 1% agarose gel electrophoresis (Figures 6–10).

The impact of the loop primers on the sensitivity of the designed kit was determined by the amplification of the template at different durations (30, 45, and 60 minutes) at 65°C using one set with loop primers, while the other set was determined without loop primers. The loop primers could reduce the amplification duration by 30 min (Figure 7(a)).

The specificity of the designed RT-LAMP kit was determined using RVFV strains commonly used in our laboratory compared with PPR and capripox viruses. The results showed that the kit was specific to the RVFV strain (Figure 7(b)). The limit of detection for the new kit was determined by analyzing the tenfold dilution of the reference RNA sample and comparing it with qRT-PCR (Figure 9). The efficiency of the qRT-PCR was established by generating a standard curve for the dilutions. The *R*^2^ and the slope were tabulated as 0.984 and −3.761, respectively (Figure 9(b)). The sensitivity of the new RVFV RT-LAMP assay was tenfold higher than that of the qRT-PCR used as a confirmatory molecular assay (Figures 9(a) and 9(c)).

Field applicability of the new colorimetric-RVFV-UDG-RT-LAMP was validated using naked eye visualization of the color change, using a colorimetric RT-LAMP following incubation at 65°C for 30 min in a water bath. For this purpose, 120 reference samples were used. The diagnostic sensitivity (DSe) and specificity (DSp) of the new kit compared to qRT-PCR and IgM ELISA were found to be 98.36% and 96.49%, respectively (Figure 10). Additionally, the positive predictive value (PPV) and negative predictive value (NPV) were found to be 96.77% and 98.21%, respectively (Figure 10).

## 6. Discussion

Rift Valley fever virus (RVFV) is a high-priority zoonotic pathogen with the ability to cause massive loss during its outbreak within a very short period of time. The choice of the RVFV *M* segment as the template for the development of the colorimetric-RVFV-UDG-RT-LAMP was based on the fact that the segment encodes mainly glycoproteins that are the major structural antigens and the most detectable part of the RVFV envelope [1, 31]. The relationship among the twenty-three RVFV M segment sequences used during alignment is shown in Figure 4. In this study, six sets of primers were successfully and skillfully designed using the NEB LAMP Primer design tool to target 211pb of the highly conserved region of the template (Table 2 and Figure 1). The selection of the NEB LAMP Primer design tool for the design of the RT-LAMP primers was based on its flexibility, precision, and worldwide acceptability [19, 32, 33]. The choice of the primer set for this study was determined by twofold factors, specificity and the ability to form a loop, as previously described [33]. The size, positions, and locations of the six designed primers are shown in (Table 2, Figures 1, and 5).

The quality and integrity of the template used in the development of any diagnostic kit are as good as the kit itself. Therefore, the emphasis on reliance on a high-quality template cannot be underestimated [34]. In this study, total RNA was extracted from Vero cells infected with RVFV showing high levels of CPE (Figure 2). The total RNA was extracted using the TRIzol reagent method. The selection of the TRIzol reagent method was based on previous studies that intimated that the TRIzol reagent method was superior to the current column-based RNA extraction kits in the generation of high-quality RNA [33, 35, 36].

The absorbance at wavelengths 260 nm, 280 nm, and 230 nm was used to determine the concentration and purity of the extracted total RNA (Table 1 and Figure 3). Since nucleic acid maximumly absorbs light at A260 nm, the concentration of total RNA extracted was automatically calculated by multiplying the absorbance at A260 nm wavelength with an extinction coefficient factor of 40 [37]. A pure RNA sample must have A260/A280 and A260/A230 ratios at 1.8–2.2 and > 1.7, respectively [36]. During this study, all values of total extracted RNA were within the recommended ratio ranges of 1.91–2.01 and 1.86–2.00 for A260/A280 and A260/A230 ratios, respectively (Table 1). These findings agree with the findings of Wang et al [36], in terms of purity. However, the concentration of extracted total RNA was found to be averagely higher compared to that extracted by Wang et al [36]. The purity and integrity of the extracted total RNA were key to downstream processing [38].

The colorimetric RVFV-UDG-RT-LAMP assay developed in this study involved four main stages: synthesis of cDNA from the RNA template, production of the starting material for the LAMP reaction, cyclic amplification, and final elongation combined with recyclization, as previously described by Sahni et al. [18]. Reverse transcription (RT) was initiated by the binding of BIP primers at the 3′ end of the RNA template in the presence of RTx reverse transcriptase present in the 2x warmstart multipurpose LAMP/RT-LAMP reaction mixed with UDG at 65°C. The B3 primer annealed to the complementary RNA sequence (B3c) at the 3′ end outside the BIP primer to initiate strand displacement DNA synthesis in the presence of bst 2.0 polymerase. This process released the BIP-linked cDNA strand. Since the sequences of B1 and B1c are complementary to one another, a loop-out structure was formed at the 5′ end. This single-stranded DNA served as a template for FIP-initiated cDNA synthesis and subsequent F3-primed strand displacement DNA synthesis. This results in two products, a double-stranded DNA segment and a single-stranded DNA sequence with F1 and F1c complementary sequences. The two sequences hybridize to form another loop at the 3′ end, forming a dumbbell-shaped structure. The dumbbell-shaped structure then served as the starting material for the loop-mediated isothermic amplification (LAMP) [17]. The third and fourth stages involve exponential and rapid amplification and reamplification of the self-priming DNA template with concomitant replacement of strands, resulting in a mixture of stem-looped DNA products of varying lengths, as shown on agarose gel electrophoresis (Figures 7–9). The findings of this study were similar to those of previous studies [17, 22, 23, 39].

The optimal amplification conditions for the colorimetric RVFV-UDG-RT-LAMP without loop primers were found to be 65°C for 60 min (Figure 6). Dao Thi et al. [39] reported findings similar to ours, contrary to the findings by Kokane et al. [22] who had established that the optimal time was 75 min. The addition of loop primers that are complementary to the loops on the dumbbell-like DNA template reduced the amplification time by 50%, from one hour to only 30 min (Figure 7(a)). These findings agreed with those of previous studies [40–42]. Lee et al. [40] and Soroka et al. [41] argued that loop primers increased the “starting points” during the LAMP reaction up to a total of eight amplified DNA sequences, thereby improving the specificity, sensitivity, and efficiency of the reaction.

In this study, the colorimetric RVFV-UDG-RT-LAMP assay developed was able to detect as low as 1.85 pg/*µ*l of total RNA extracted from the RVFV-infected cells within 30 min at 65°C (Figure 7(a)). The inclusion of uracil-DNA glycosylase (UDG) enzyme in the RT-LAMP reaction mix prevented carry-over contamination by the termination of any nonspecific amplification during the RT-LAMP reaction [43, 44]. This was based on previous studies that established that UDG hydrolyzes the N-glycosidic bond between deoxyribose sugar and uracil in DNA containing deoxyuridine in place of thymidine by initiating the base excision repair (BER) pathway [44]. This process removes uracil resulting from spontaneous deamination of cytosine or incorporation of dUMP during DNA synthesis [44]. However, since UDG could not remove preexisting contamination from standard dTTP-containing PCR products, good laboratory practices were employed and the RT-LAMP reagents were prepared in a separate sterile room from where the samples were kept and/or added to the reaction mix [43].

The RVFV-UDG-RT-LAMP amplification products were detected through agarose gel electrophoresis and turbidimetric and colorimetric analyses (Figures 6–9). In this study, gel electrophoresis was only limited to the optimization of the amplification parameters due to the limitations associated with the time of preparing the gel and electrophoresis process and the inclusion of a visualization dye, ethidium bromide, which is carcinogenic [41]. Unlike the agarose gel images showing only one distinct band during gel electrophoresis of PCR for positive results, in the RT-LAMP reaction, bands of different lengths were observed (Figures 6, 7, and 9). Silva et al. [45], attributed this to the fact that there are different start points and hence different lengths of DNA template products.

Turbidimetric analysis was achieved by the observation of a white precipitate after a brief spin (Figure 8). The white deposit was magnesium pyrophosphate (Mg_2_P_2_O_7_), a byproduct of the LAMP reaction [46, 47]. During RT-LAMP reaction, pyrophosphate is formed, which in turn, reacts with the magnesium ions present in the reaction buffer, creating Mg_2_P_2_O_7,_ a white sediment that is visible after a brief centrifugation [48]. Yuan et al. [21] and Silva et al. [45], reported similar findings in their previous studies. However, this is highly dependent on individual judgment, making it unreliable in cases where the product is low in quantity.

Colorimetric analysis of the RT-LAMP assay products was achieved by the addition of phenol red in the Warmstart LAMP 2x master mix with UDG. This made it easier to identify positive amplification by observing a color change because of the change in pH during amplification from basic to acidic [49]. Phenol red has a red color in the basic environment and turns yellow in the acidic environment. Therefore, tubes whose reaction content color turned yellow were termed positive while those that remained red were termed negative (Figures 7 and 9). Color changes correlated well with the agarose gel electrophoresis results, providing a quicker and safer way of identifying the RT-LAMP reaction results (Figures 7(b) and 9(c)). These findings agree with the findings of previous studies by Poole et al. [19] and Zhang et al. [33].

In this study, samples selected for validation of the new kit were first subjected to commercial IgM capture ELISA. Both ELISA positive and negative samples were selected and subjected to qRT-PCR and colorimetric-RVFV-UDG-RT-LAMP. This is because IgM antibodies to RVFV last only for six to eight weeks after the initial exposure to the RVFV and their presence indicates recent or current RVFV infection [50, 51]. The colorimetric-RVFV-UDG-RT assay was found to be ten-fold more sensitive compared to the qRT-PCR (Figures 9(a) and 9(c)). These findings agreed with the findings of Han et al. [14], Poole et al. [19], and Marino et al. [52], who established that the RT-LAMP assays are more sensitive to the detection of lower concentrations of target organisms, making them ideal for field/point-of-care diagnostics. The diagnostic sensitivity (DSe) and specificity (DSp) of the designed kit were found to be 98.36% and 96.49%, respectively. Additionally, the PPV and NPV were found to be 96.77% and 98.21%, respectively (Figure 10). Similar findings were previously reported [39, 44, 49]. However, the DSe and DSp found in this study were slightly lower than those established by Asih et al. [53]. The DSe and DSp were used to assess the ability of the designed kit to correctly classify a sample as positive and negative, respectfully [44]. Since the DSe and DSp may be misleading due to the false positive and false negative, it was paramount to establish the positive predictive value (PPV) and negative predictive value NPV [54]. The PPV and NPV take into account the false positives and false negatives and demonstrated the practical usefulness of the new kit in screening the RVFV samples in the field [55].

## 7. Conclusions and Recommendations

The colorimetric RVFV-UDG-RT-LAMP assay developed could positively detect RVFV in RNA extracted by MagMAX viral RNA isolation kit. Both blood and serum samples could be used as samples. The new kit would take a minimum of 30 min at 65°C to obtain the results. There was a 100% correlation between color change and positive amplification. Importantly, our colorimetric RVFV-UDG-RT-LAMP assay yielded comparable diagnostic results to qRT-PCR when directly applied to RNA samples. Further studies need to be carried out to develop a lateral flow assay based on this RT-LAMP principle to improve the efficiency and usability of the new kit for the diagnosis of RVFV.

## Figures and Tables

**Table 1:** Concentration and purity of total RNA extracted from the monolayer of Vero cells infected by different viruses using TRIzol reagent (group means ± SD).

	Concentration (ng/*µ*L)	Purity	
	Spectrophotometer (*n* = 3)	OD 260/280	OD 260/230
Uninfected Vero cells	1421.27 ± 4.27	1.91 ± 0.08	1.86 ± 0.04
RVF 5335	4643.70 ± 27.24	1.93 ± 0.04	1.90 ± 0.04
PPR virus	4067.57 ± 34.71	1.98 ± 0.13	1.97 ± 0.04
RVF G64	3458.97 ± 13.86	2.01 ± 0.09	1.83 ± 0.06
RVF 1983	3432.07 ± 8.32	1.96 ± 0.06	2.00 ± 0.04
RVF G66	2944.40 ± 55.45	1.99 ± 0.02	1.95 ± 0.07

**Table 2:** Details of the RT-LAMP Glycoprotein gene primer set for rapid and real-time detection of RVFV.

Primer's name and description	Position on the genome^a^	Length of the oligonucleotide (bp)	Oligonucleotide sequence
F3	2584–2602	19	GGGTGCATAAACTCACTCT
B3	2775–2794	20	CGAGGAATTTCTGAGAATGG
FIP (F1c + F2)	2667–2635 2615–2652	42	GCTGACTGAACCCCAGTTTGTCTTTGATGGCTCTGTTTCAAC
BIP (B1c + B2)	2696–2715	40	GGACGCAGAGGGCATTTCAGCATCAACAATTGCATACCCT
	2753–2773		
LF	2636–2656	21	GATGATGCTCCCAAGTCTACT
LB	2731–2752	22	CTTTCATTGAGAGCCCAGGCAA
F2	2615–2652	21	CTTTGATGGCTCTGTTTCAAC
B2	2753–2773	20	CATCAACAATTGCATACCCT
F1c	2667–2635	21	GCTGACTGAACCCCAGTTTGT
B1c	2696–2715	20	GGACGCAGAGGGCATTTCAG

^a^RVFV ZH-501 strain (genebank accession code: M11157.1).

## Data Availability

All data and material that support the findings of this study are included in this manuscript.
